# The added value of post-mortem MRI in forensic imaging: an observational study in the Netherlands

**DOI:** 10.1186/s13244-026-02418-x

**Published:** 2026-10-15

**Authors:** Cédrique A. S. Gregoire, Maartje M. A. Eggen, Geneviève A. J. C. Crombag, Alida A. Postma, J. Martijn Nobel

**Affiliations:** 1 Department of Radiology and Nuclear Medicine, Maastricht University Medical Centre+ (MUMC+), Maastricht, The Netherlands; 2 Care and Public Health Research Institute (CAPHRI), Maastricht University, Faculty of Health, Medicine and Life Sciences, Maastricht, The Netherlands; 3 Mental Health and Neuroscience Research Institute (MHeNs), Maastricht University, Faculty of Health, Medicine and Life Sciences, Maastricht, The Netherlands; 4 Research Institute for Oncology and Reproduction (GROW) School for Oncology and Reproduction, Maastricht University Medical Centre+, Maastricht, The Netherlands

**Keywords:** Forensic imaging, Postmortem imaging, Whole body imaging, Cause of death, Crime

## Abstract

**Objectives:**

In the Netherlands, post-mortem radiology aids forensic investigation, using a full-body post-mortem computed tomography (PMCT) scan and a post-mortem magnetic resonance imaging (PMMRI) scan in specific cases. Application of PMMRI varies between countries and institutions in the absence of international guidelines. This study analyses current use and added value of PMMRI in a single forensic imaging institution in the Netherlands.

**Materials and methods:**

All post-mortem cases scanned between July 2024 and July 2025 were included, except non-accidental injury protocol cases. The (contra-)indications for additional PMMRI scans in addition to PMCT were recorded. On the same day as image evaluation, added value was scored by the forensic radiologist and consultant using the following options: additional finding, exclusion of a finding, confirmation of PMCT and no added value.

**Results:**

PMMRI was conducted in 47.4% of all cases, indicated by suspected strangulation, penetrating injuries, blunt force trauma and lesions seen on PMCT. In suspected strangulation, PMMRI neck showed additional findings or exclusion of findings in 31/46 cases, while PMMRI brain had the lowest added value in 13/25 cases. PMMRI thorax and abdomen showed additional findings in 14/20 and 4/9 cases of penetrating trauma, respectively. Additional findings were found in 10/15 PMMRI brain scans and 6/8 PMMRI thorax scans, based on PMCT.

**Conclusion:**

Application of PMMRI in addition to PMCT is currently used in a targeted manner. In cases of strangulation and penetrating injuries, additional or excluded findings were found. Therefore, adding PMMRI in these specific cases can be of added value.

**Key Points:**

***Question*** What is the added value of post-mortem MRI in forensic cases, in the absence of international guidelines and standardised indications?

***Findings*** Additional findings were observed in post-mortem MRI examinations indicated for soft tissue injuries caused by penetrating force and in cases of suspected strangulation.

***Critical Relevance Statement*** The criminal investigation can be optimised by using post-mortem MRI in addition to post-mortem CT, either by showing additional findings on post-mortem MRI not seen on CT, or by excluding findings with higher certainty.

**Graphical Abstract:**

**Figure:**
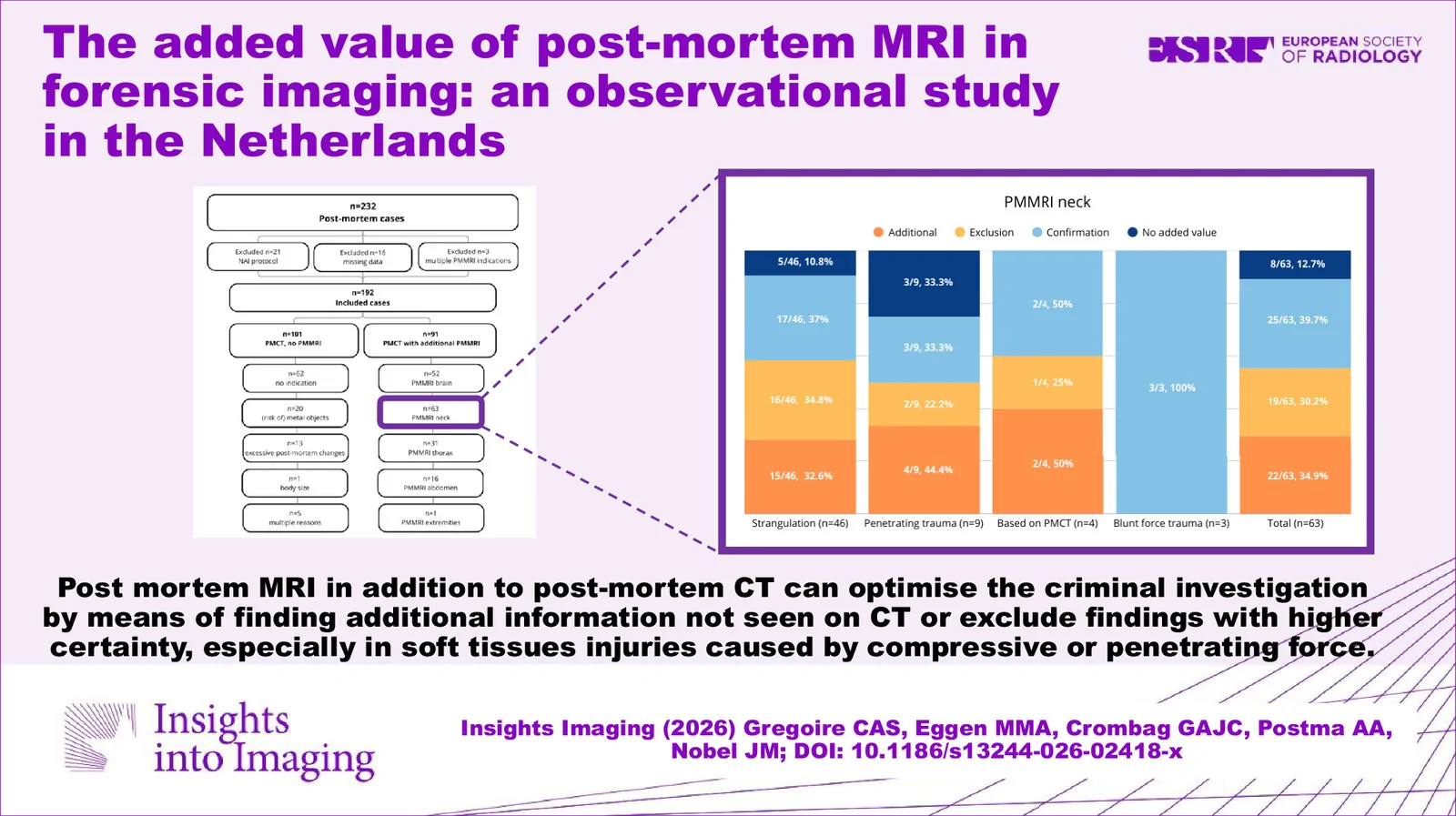

## Introduction

Under Dutch law, a distinction between a natural and unnatural manner of death is made. The public prosecutor may request further post-mortem examination, including radiology, toxicology and autopsy, in case of an unnatural manner of death or if homicide, suicide or an accident cannot be ruled out [1–3]. This forensic investigation differs from a clinical setting, in which post-mortem examination can be requested by the family to establish the cause of death [3]. To determine whether a criminal offence has occurred, it is important to recognise which radiological diagnostics—“Post-Mortem Computed Tomography (PMCT)”, “Post-Mortem Magnetic Resonance Imaging (PMMRI)” and, in children, skeletal survey (X-rays)—are indicated in which circumstances. Since “Post-Mortem Computed Tomography Angiography (PMCTA)” is not used in Dutch practice, PMCTA is not part of the forensic diagnostic tools in the Netherlands [4].

Within post-mortem investigations, literature shows that PMMRI is superior at visualising soft tissue and organ lesions compared to PMCT [5–12]. Evaluating the brain, PMMRI is more accurate in detecting intra- and extra-axial haemorrhage and parenchymal injury, e.g. infarction, coup-contrecoup lesions and “Diffuse Axonal Injury (DAI)” [9, 13–17]. PMMRI has an advantage in detecting compressive forces on the neck, including subcutaneous swelling and haemorrhage in neck muscles, lymph nodes, and tissue around fractures [9, 18–21]. Regarding the thorax, PMMRI can be able to depict myocardial infarction and pulmonary embolisms as well as organ injuries due to blunt force or penetrating trauma, albeit in daily practice still challenging [5, 9, 22–28]. Most life-threatening abdominal injuries can be visualised using PMMRI [5, 9, 29]. For skeletal trauma, CT is superior to PMMRI for fracture detection, although PMMRI can provide insights into bone bruises by the presence of bone marrow oedema [5, 30, 31].

While PMCT is widely implemented in forensic investigations, the use of PMMRI differs greatly between countries and institutions, and no (inter)national guidelines have been developed yet [5, 32, 33]. An important burden in forensic cases for using PMMRI is the presence of metallic objects, advanced decomposition and more logistical challenges, such as the availability of scanners and associated expertise, including experienced technicians and radiologists [5, 34]. However, PMMRI has been studied increasingly in recent years, raising questions about its potential and in which cases it may offer added value [33, 34]. In terms of radiological assessment, this may involve additional findings, ruling out findings and increasing certainty of findings on PMCT.

In the Netherlands, radiological investigation requested by the public prosecutor includes a total body PMCT in all adult cases followed by an additional PMMRI in certain cases. In this study, the use of PMMRI during one year is analysed to identify prevalent (contra)indications and to determine the added value from the radiologist’s point of view in addition to PMCT. Given the heterogeneous global applications of PMMRI in the forensic setting, this study aims to identify potentially standardisable indications.

## Materials and methods

This study was legally approved by the Netherlands Public Prosecution Service, and the local Medical Ethical Committee confirmed that informed consent was waived (METC 2025-0294).

This observational study was conducted at an academic medical centre in the Netherlands. All forensic post-mortem cases, scanned in one of the affiliated locations between July 2024 and July 2025, were included. Cases scanned in the context of the “Non-Accidental Injury (NAI)” protocol in children were excluded, since there is an (inter)national consensus on the value of PMMRI in these cases [35–38]. Additionally, cases in which PMMRI has been performed based on multiple indications (e.g. PMMRI neck indicated on both strangulation and penetrating injury) were excluded.

In all included cases, a full-body PMCT was performed, supplemented by PMMRI in specific cases. All cases were scanned in a body bag in supine position. PMCT was performed using either a NAEOTOM Alpha photon-counting (Siemens Healthineers), a SOMATOM Force (Siemens Healthineers), or an Acquilion ONE (Canon Medical Systems) CT scanner. Images were reconstructed at a maximum 1.0 mm slice thickness using both soft and sharper kernels for optimal visualisation of soft tissues and bone. PMMRI images were acquired using 1.5-Tesla MRI (Ingenia Ambition S, Philips Health Systems) or 3-Tesla MRI (Ingenia CX/7700, Philips Health Systems). To assess water-rich structures, T2-weighted sequences are used, with fat suppression and an iron deposition-specific sequence (FFE or SWI) if necessary. The scanning protocol can be found in Table 1, whereby additional sequences could be added.

**Table 1:** Scanning protocol per body part-specific PMMRI^*^

PMMRI brain	PMMRI neck	PMMRI thorax/abdomen	PMMRI extremities
Axial FLAIR (3 mm)	Axial STIR (3 mm)	Axial T2 TSE (4 mm)	Axial T2 TSE (4 mm)
Axial T2 TSE (3 mm)	Axial T2 TSE (3 mm)	Sagittal T2 TSE (4 mm)	Axial T1 (4 mm)
Axial T2 FFE (3 mm)	Axial T2 FFE (3 mm)	Coronal T2 TSE (4 mm)	
3D T1 FFE (1 mm)	Axial T1 (3 mm)	Axial T1 (4 mm)	
Coronal IR (3 mm)	Coronal STIR (3 mm)	Axial T2 FFE (4 mm)	
DWI b600 (3 mm)		3D T2 TSE (1 mm)	
DWI b1000 (3 mm)			

^*^ Specific settings depend on the temperature of the body

All images were evaluated by one of seven radiologists (1–15 years’ experience in forensic and post-mortem radiology) and one of four forensic radiology consultants (being a medical doctor (1–3 years forensic imaging experience) or a forensic technician (> 8 years forensic imaging experience), using dedicated radiological software (Sectra IDS7 image viewer, Sectra AB). Under applicable legal provisions, this early phase yields a strictly factual report without interpretation. The decision to add a PMMRI scan was made by the evaluating radiologist and consultant depending on the case circumstances or based on findings of the full body PMCT. Upon request, the consultant received available background information necessary for the scanning procedure; the radiologist remained blinded to this information during the procedure. According to the operating method, PMMRI brain and neck were performed in cases of suspected force impact on the neck (e.g. strangulation, hanging, or smothering). Additionally, a body part-specific PMMRI was conducted in cases of penetrating trauma (e.g. shooting or stabbing), blunt force trauma or when a lesion of interest was seen on PMCT.

In this one year, all (contra)indications and the value of the added PMMRI were scored, using pre-determined options (see Table 2). Exclusion of a finding refers to exclusion of a diagnosis rather than the CT appearance itself. Absolute contraindications included (suspected) presence of metallic objects (e.g. projectiles or small fragments of potential forensic relevance like a knife blade fragment) and advanced post-mortem changes resulting in extensive air artefacts. Before starting this cohort study, a plenary explanation was given to the team at the start of the inclusion period. The added value was scored ‘per finding’, which means that one PMMRI scan can show both an additional finding and the exclusion of another. The radiologist and consultant then recorded what these findings entailed. The (contra)indication(s) were verified in retrospect, as this information is stated in the completed (application) forms. Cases which lacked a value score (missing data) were excluded, since this entailed a subjective scoring at the time of assessment of the images, and retrospective analysis was therefore not eligible. When there were multiple PMMRI scans conducted and the recorded value score was incomplete (e.g. a PMMRI brain and neck in a suspected strangulation case, where only the added value of the PMMRI neck was recorded), the assumption was made that the scan that was not mentioned, had no added value. Correction of this assumption can be found in a sensitivity analysis in Table S1.

**Table 2:** Scoring system (contraindications), indications and value PMMRI

No PMMRI	**Contra-indications**	
	No indication based on the case circumstances and PMCT	
	(Risk of) metals in the body	
	Excessive post-mortem changes	
	Other	
PMMRI per body part	**Indications**	**Added value scoring**
	Suspected strangulation	Additional finding
	Blunt force trauma	Exclusion of a finding
	Penetrating injury	Confirmation of PMCT
	PMMRI based on PCMT	No added value
	Other	

Data were collected using Microsoft Excel 2016. Descriptive statistics were used to analyse the (contra)indications per body part. Secondly, the added value was analysed per indication and body part. Due to low incidence numbers, indications with an *n* < 3 were excluded from this second analysis. For all body parts, the complete table, including 95%- confidence interval, can be found in Table S2.

## Results

**Fig. 1:**
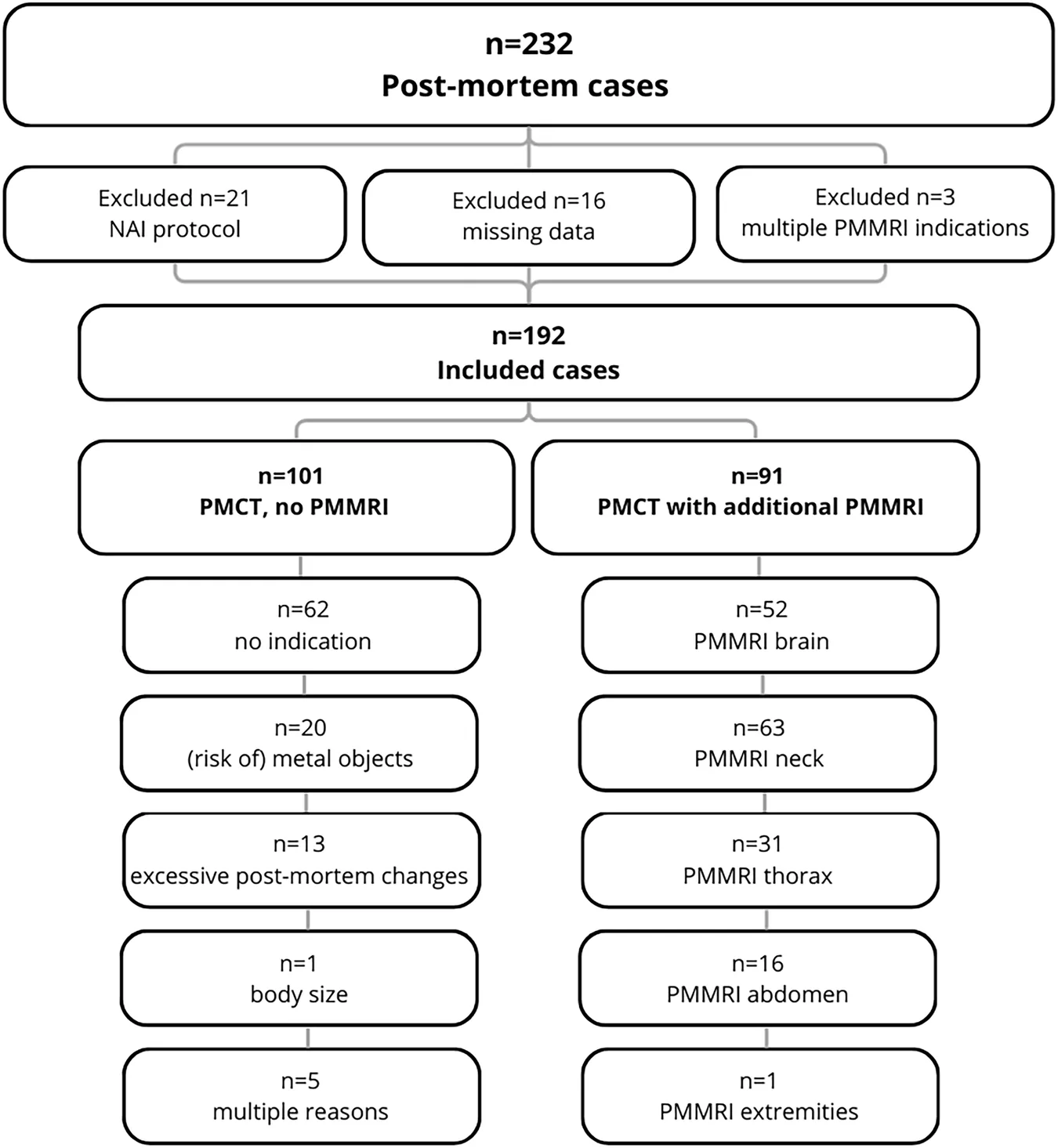
Flowchart of the case selection process

**Fig. 2:**
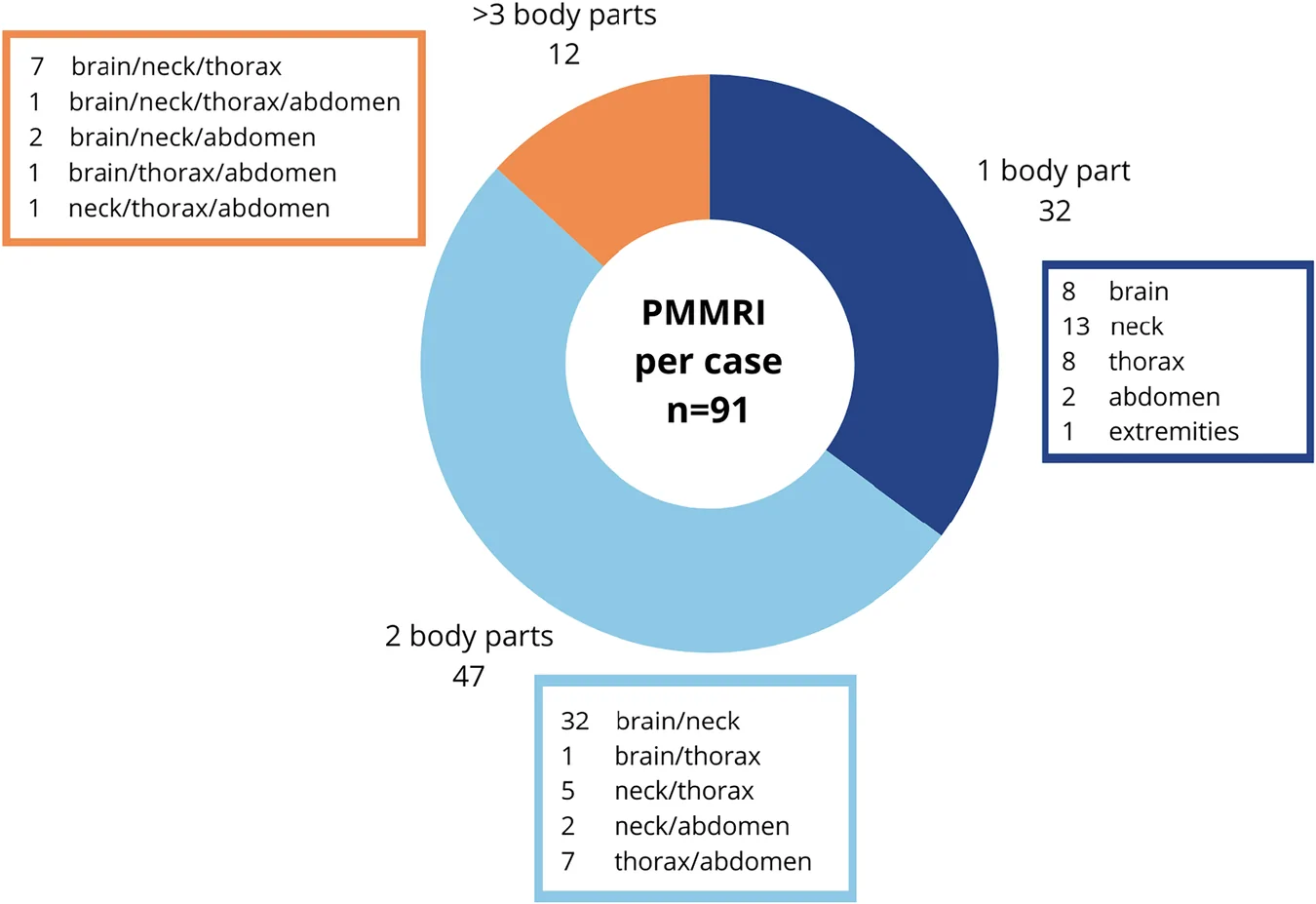
Number of PMMRI scans with corresponding body part per case

Of 232 post-mortem cases between July 2024 and July 2025, a total of 40 cases were excluded: 21 NAI protocol cases, 16 cases of missing data and 3 cases with multiple PMMRI indications. This left 192 included cases, of which in 101 cases (52.6%) no additional PMMRI was made based on: no indication (61.4%), (risk of) metal objects (19.8%), extensive post-mortem changes (12.9%), body size (1%) or multiple of the aforementioned reasons (5%) (Fig. 1). In 91 cases an additional PMMRI was performed, accounting for 163 PMMRI-scans: brain (*n* = 52), neck (*n* = 63), thorax (*n* = 31), abdomen (*n* = 16) and extremities (*n* = 1) (Fig. 1). In 32 cases one body part was scanned, in 47 cases 2 body parts were scanned and 3 or more body parts were scanned in 12 cases (Fig. 2).

### PMMRI brain

**Fig. 3:**
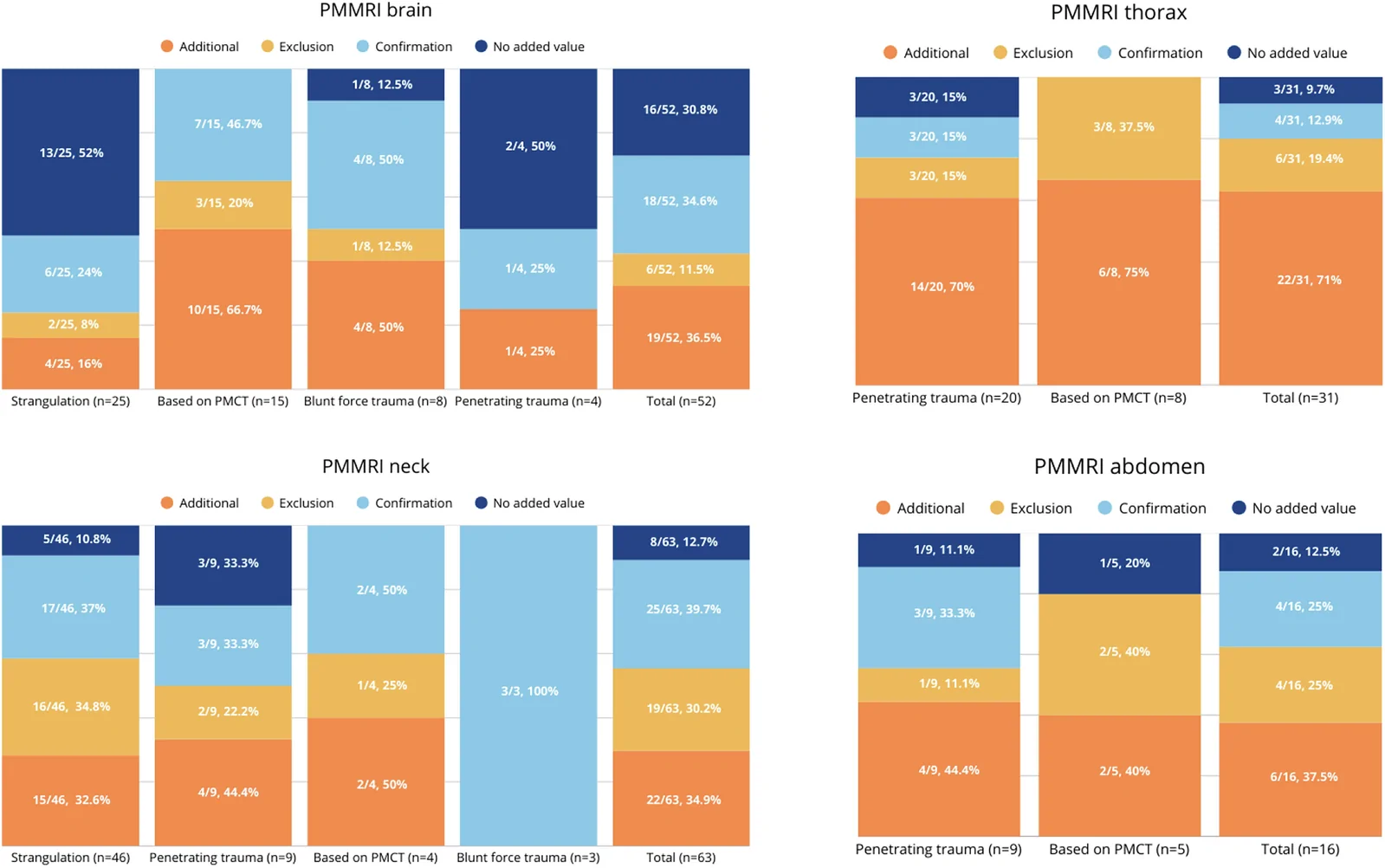
Added value per body part-specific PMMRI and per indication. In this figure, the added value score (additional finding, exclusion of a finding, confirmation of PMCT, and no added value) of the body part-specific PMMRI is shown per indication. For each score, the ratio to the number of PMMRI scans is shown with the corresponding percentage. Each PMMRI was scored per finding, which may result in more findings being reported than scans. Only indications (*n* > 3) are included for analysis in this figure, explaining the differences in totals

Indications for 52 PMMRI brain scans included suspected strangulation (*n* = 25), PMMRI based on PMCT (*n* = 15), blunt force trauma (*n* = 8) and penetrating injury (*n* = 4) (Table 3). In 25 suspected strangulation cases, four additional findings were found (16%, extra-axial haemorrhage, brain herniation, subcutaneous swelling or not further specified) and 13 scans showed no added value (52%) (Fig. 3). In 10/15 cases of PMMRI based on PMCT, additional findings were identified (66.7%, brain herniation, parenchymal oedema or injury, intra-axial haemorrhage, subcutaneous swelling, an aneurysm and better visualisation of the lesions seen on PMCT) and zero cases showed no added value (Fig. 3). In blunt force trauma cases 4/8 additional findings were detected (50%, subcutaneous swelling, intra- and extra-axial haemorrhage and parenchymal injury) and one case showed no added value (Fig. 3). Finally, one additional finding was seen in four cases of penetrating injury (25%, better visualisation of the trajectory) and two cases showed no added value (50%) (Fig. 3).

**Table 3:** Indications for PMMRI per body part

PMMRI per body part, *n* = 163	Indication	Total (*n*, %)
PMMRI brain	Total	52/163 (31.9%)
	Suspected strangulation	25/52 (48.1%)
	PMMRI based on PMCT	15/52 (28.8%)
	Intra- or extra-axial haemorrhage	10/52 (19.2%)
	Cerebral oedema	3/52 (5.8%)
	Cerebral infarction	1/52 (1.9%)
	Spinal cord injury	1/52 (1.9%)
	Blunt force trauma	8/52 (15.4%)
	Penetrating injury	4/52 (7.7%)
PMMRI neck	Total	63/163 (38.7%)
	Suspected strangulation	46/63 (73.0%)
	Penetrating injury	9/63 (14.3%)
	PMMRI based on PCMT	4/63 (6.4%)
	Spinal cord injury	2/63 (3.2%)
	Carotid artery dissection	1/63 (1.6%)
	Difficulty interpreting due to cachexia	1/63 (1.6%)
	Blunt force trauma	3/63 (4.8%)
	Suspected oesophageal injury	1/63 (1.6%)
PMMRI thorax	Total	31/163 (19.0%)
	Penetrating injury	20/31(64.5%)
	PMMRI based on PMCT	8/31 (25.8%)
	Pleural effusion	3/31 (9.7%)
	Aortic abnormality	3/31 (9.7%)
	Difficulty interpreting due to cachexia	1/31 (3.2%)
	Spinal cord injury	1/31 (3.2%)
	Blunt force trauma	2/31 (6.5%)
	Suspected oesophageal injury	1/31 (3.2%)
PMMRI abdomen	Total	16/163 (9.8%)
	Penetrating injury	9/16 (56.3%)
	PMMRI based on PMCT	5/16 (31.3%)
	Abdominal effusion	3/16 (18.8%)
	Aortic injury	1/16 (6.3%)
	Difficulty interpreting due to cachexia	1/16 (6.3%)
	Suspected early pregnancy	2/16 (12.5%)
PMMRI extremities	Total	1/163 (1.6%)
	PMMRI based on PMCT: vascular injury	1/1 (100%)

### PMMRI neck

PMMRI neck scans (*n* = 63) were indicated on: suspected strangulation (*n* = 46), penetrating injuries (*n* = 9), PMMRI based on PMCT (*n* = 4), blunt force trauma (*n* = 3) and suspected oesophageal injury (*n* = 1) (see Table 3). In 15/46 cases of suspected strangulation, additional findings were detected (32.6%, swelling or haemorrhage in the neck and brain stem), and in 16/46 cases findings could be excluded (34.8%, exclusion of injury of the neck and vascular injury). Five PMMRI scans showed no added value in suspected strangulation (10.9%) (Fig. 3). Regarding penetrating injuries, in 4/9 cases additional findings were detected (44.4%, better visualisation of the trajectory, skin and soft tissue defects, vascular injury and an injury to the vocal cords) and three cases showed no added value (33.3%). Two additional findings were identified out of four cases of PMMRI based on PMCT (50%, subcutaneous swelling and better differentiation of spinal cord injury), and zero cases showed no added value. In blunt force trauma, all findings confirmed PMCT (3, 100%, not further specified) (Fig. 3).

### PMMRI thorax

As presented in Table 3, indications for PMMRI thorax scans (*n* = 31) consisted of penetrating injury (*n* = 20), PMMRI based on PMCT (*n* = 8), blunt force trauma (*n* = 2) and suspected oesophageal injury (*n* = 1). In 14/20 cases of penetrating injury, additional findings were found (70%, cardiac-, lung- and thoracic wall injury, haematoma and better visualisation of the trajectory), and three cases showed no added value (15%) (Fig. 3). In 6/8 cases of PMMRI based on PMCT, additional findings were identified (75%, differentiation of pleural effusion, myocardial infarction, aortic dissection, spinal cord injury and subcutaneous swelling), and no cases showed no added value (Fig. 3).

### PMMRI abdomen

Indications in 16 PMMRI abdomen scans included penetrating injury (*n* = 9), PMMRI based on PMCT (*n* = 5) and suspected early pregnancy (*n* = 2) (Table 3). In 4/9 cases of penetrating injury, additional findings were detected (44.4%, liver injury, spinal cord injury and differentiation of abdominal effusion), and one case showed no added value (Fig. 3). In 4/5 cases of PMMRI based on PMCT, additional findings or exclusion of findings were determined (80%, differentiation of abdominal fluid and aortic injury), and one case showed no added value (Fig. 3).

### PMMRI extremities

One PMMRI extremities scan was performed in a case based on potential vascular injuries on PMCT. The PMMRI scan confirmed the findings seen on PMCT.

## Discussion

In this observational cohort study, 192 cases were evaluated over one year to assess (contra)indications for PMMRI as an adjunct to PMCT, and in 91 cases the added value of PMMRI was analysed. Added value was scored in 163 body-part-specific PMMRI scans by the evaluating radiologist and consultant based on four characteristics: additional finding, exclusion of a finding, confirmation of PMCT or no added value. In 52.6% of the cohort, no PMMRI was performed, of which in 61.4% no indication was seen, indicating a certain reluctance in the use of PMMRI already. The most common indications for a body-part-specific PMMRI included strangulation (*n* = 71), penetrating injury (*n* = 42) and blunt force trauma (*n* = 13), reflecting the current operating method. Yet, PMMRI based on PMCT (*n* = 33) was a frequent indication as well.

### (suspected) Strangulation

All cases with strangulation suspicion were given an additional PMMRI neck, but only 25/46 received a PMMRI brain. This was possibly due to negative findings seen on the initial PMCT brain and the urge to rule out pathology in the neck region with increased certainty. A high positive value was found in the application of PMMRI neck in suspected strangulation (additional finding or exclusion of a finding in 31/46 cases). This is supported by literature, which shows that PMMRI enhances the radiological examination by PMCT in detecting haemorrhages [18–21]. Since both the presence and absence of findings aid in evaluating cases of possible strangulation, PMMRI can provide more detail on the soft tissue surrounding the larynx, thereby increasing certainty in the overall assessment combined with PMCT. A recent review on strangulation indicators states absence of external injuries is up to 16% in fatal cases, and also in non-fatal cases a considerable number of individuals have no visible injuries [39–41]. Therefore, it may be valuable to use PMMRI even in cases of low to no suspicion of strangulation to detect any (subtle) internal abnormalities.

Low added value for PMMRI brain was found, as shown by a low rate of additional findings (4/25 cases) and a high rate of no added value (13/25 cases). Signs of fatal asphyxia and consequent brain hypoxia can be expected in strangulation cases, but this depends on the type of strangulation and pathophysiology [20, 42–44]. Clinical research has shown that the sensitivity of MRI in detecting hypoxia is significantly higher than CT, yet partially depends on the timing after the event, and that structural appearance of hypoxia may be a strangulation indicator in non-fatal cases [43–45]. Rapid death after strangulation might leave insufficient time for post-anoxic brain injuries to become visible on PMCT and PMMRI, which could explain the low added value found in this study and the limited effects on the brain in other studies after fatal strangulation [19, 20].

### Penetrating injuries

Penetrating injuries -stabbing and shooting incidents- accounted for 25.8% of all PMMRI scans. Added value was found by additional findings in the neck, thorax and abdomen (respectively, 4/9, 14/20 and 4/9 cases). The common denominator in this added value was better appreciation of soft tissue and organ lesions, as observed in the literature as a rationale for PMMRI application [5, 9, 46]. Whereas PMCT might indicate suspected injury by, e.g. a blood layer surrounding the affected organ, PMMRI often proves capable of confirming this and assessing it more precisely. Even though PMMRI provides higher contrast in soft tissue structures, it remains difficult to detect vascular lesions in frequently collapsed post-mortem blood vessels. Depending on the local workflow, PMCTA can be considered for the assessment of the potential bleeding source, however, this technique is not part of the radiological diagnostic tools in post-mortem imaging in the Netherlands [4, 47]. As a result of better tissue contrast on PMMRI, more detailed assessment of the injuries can lead to a better visualisation and more precise determination of the trajectory in case of penetrating injuries. As such, additional findings in this cohort represented mainly solid organs, such as the heart and liver (respectively, 70% and 44%).

Minor added value (50% no added value) was found in cases of penetrating injury of the head. This could potentially be due to a small amount of soft tissue on the skull and the low incidence rate of penetrating injury in the head, compatible with the number of PMMRI scans in our study (*n* = 4).

### Blunt force trauma

In cases of blunt force trauma, most PMMRI scans were performed for the brain (*n* = 8) and neck (*n* = 3), with additional findings in 4/11 cases and confirmation of PMCT findings in 7/11 cases. All additional findings relate to soft tissue lesions in the head, regarding the subcutis, brain parenchyma or haemorrhages, for which the relevance of MRI is recognised in the literature [9, 13–16]. Especially fluid inversion (FLAIR) and susceptibility-sensitive (SWI) sequences seem to have added value in cases involving more subtle traumatic brain injury, in which PMCT is inferior in detecting microbleeds and DAI [5, 48, 49]. To ensure adequate post-mortem FLAIR imaging, the inversion time requires case-specific adjustment [50, 51].

### PMMRI based on PMCT

A considerable part of all body part-specific PMRI scans were indicated based on findings on PMCT (20.2%), mainly in the brain and thorax. High added value was found, shown by additional findings in 20/33 and exclusions in 9/33 scans. This implies that when PMMRI is requested on professional grounds - following the evaluation of PMCT—it offers added diagnostic value in the majority of cases, beyond merely increasing diagnostic confidence. Again, additional findings are observed in soft tissue lesions, including brain lesions, vascular abnormalities such as aneurysm and dissection, myocardial infarction and spinal cord injury. Also, T1- and T2-weighted sequences made differentiation between haemorrhagic and other fluid collections possible in thoracic and abdominal fluid collections, inconclusive using only PMCT [52, 53]. In cases without penetration, strangulation or blunt force trauma indications, such findings may contribute to the distinction between a natural and unnatural manner of death.

### Limitations and future directions

First, the relatively large size of the scoring team and the single explanation of the scoring method, could have led to scoring differences and possibly to missing data. Although radiologists were initially blinded, same-day assessment by the same observers can potentially introduce observer bias. In addition, as the four added value scoring options can sometimes overlap in specific cases, this could introduce ambiguity, as in such cases one could select one or several options. Furthermore, the confirmation of the PMCT option is difficult to interpret, since these findings were rarely specified and therefore it was not possible to state forensic relevance in these cases. Finally, the educated assumption was made that when the added value score was incomplete, there was no added value for the missing scan. As such, these limitations could have led to an underestimation of the outcomes rather than an overestimation of findings. In this study, no indications were found apparent to warrant performing an additional PMMRI in 32% of the cohort. It may be interesting for future research to investigate whether there is added value in this group after all. Furthermore, it is important to further investigate the degree of diagnostic certainty in the radiological assessment of a forensic case by adding PMMRI. To adequately assess the case, the criminal investigation must be considered in its entirety to determine whether PMMRI provides added value. In particular, it is important to take contextual and other medicolegal findings into account, in accordance with local workflow and applicable legal frameworks.

## Conclusion

This observational cohort study demonstrates that PMMRI is currently used in a targeted manner in the Netherlands in addition to PMCT in cases of an (suspected) unnatural manner of death. Added value by additional or excluded findings is found, especially in cases of suspected strangulation, but also in cases with soft tissue injury, organ trajectories and lesions seen on PMCT.

## Supplementary information

ELECTRONIC SUPPLEMENTARY MATERIAL

## Data Availability

The dataset is not publicly accessible due to privacy and legal considerations given criminal casework. Availability needs to be conferred with the Netherlands Public Prosecution Service.

## Abbreviations

DAI: Diffuse axonal injury
NAI: Non-accidental injury
PMCT: Post-mortem computed tomography
PMMRI: Post-mortem magnetic resonance imaging
