# Supplementary material for: The added value of post-mortem MRI in forensic imaging: an observational study in the Netherlands
Source: Insights Imaging. 2026 Oct 15;17:258. doi: 10.1186/s13244-026-02418-x (PMC13672765; doi:10.1186/s13244-026-02418-x)
Supplement: Supplementary file 1 [file 13244_2026_2418_MOESM1_ESM.pdf]

The added value of Post-Mortem MRI in forensic imaging: an observational study in the Netherlands

ELECTORNIC SUPPLEMENTARY MATERIAL

Table S1 Sensitivity analysis per body-part specific PMMRI corrected for the no-added value assumption

PMMRI per body part (n=163)	Correction	Additional finding	Exclusion of a finding	Confirmation of PMCT	No added value
		(n, %, CI 95%)			
**PMMRI brain (n=52)**	Assumption if not stated, no added value	19/52 (36.5%, 24.8 – 50.1)	6/52 (11.5%, 5.4 – 22.9)	18/52 (34.6%, 23.2 – 48.2)	16/52 (30.8%, 19.9 – 44.3)
	Assumption corrected, missing data (n=15)	19/37 (51.4%, 35.9 – 66.6)	6/37 (16.3%, 7.7 – 31.3)	18/37 (48.6%, 33.5 – 64.1)	1/37 (2.7%, 0.5 – 13.8)
**PMMRI neck (n=63)**	Assumption if not stated, no added value	22/63 (34.9%, 24.3 – 47.3)	19/63 (30.2%, 20.2 – 42.4)	25/63 (39.7%, 28.5, 52.0)	8/63 (12.7%, 6.6 – 23.1)
	Assumption corrected, missing data (n=6)	22/57 (38.6%, 27.1 – 51.6)	19/57 (33.3%, 22.5 – 46.3)	25/57 (43.9%, 31.8 – 56.7)	2/57 (3.5%, 0.9 – 11.9)
**PMMRI thorax**	Assumption if not stated, no added value	22/31 (71%, 53.4 – 83.9)	6/31 (19.4%, 9.2 – 36.3)	4/31 (12.9%, 5.1 – 28.9)	3/31 (9.7%, 3.4 – 24.9)

**(n=31)**	Assumption corrected, missing data (n=3)	22/28 (78.6%, 60.5 – 89.8)	6/28 (21.4%, 10.2 – 39.5)	4/28 (14.3%, 5.7 – 31.5)	0/28 (0%)
**PMMRI abdomen (n=16)**	Assumption if not stated, no added value	6/16 (37.5%, 18.5 – 61.4)	4/16 (25%, 10.2 – 49.5)	4/16 (25%, 10.2 – 49.5)	2/16 (12.5%, 3.5 – 36.0)
	Assumption corrected, missing data (n=2)	6/14 (42.9%, 21.4 – 67.4)	4/14 (28.6%, 11.7 – 54.7)	4/14 (28.6%, 11.7 – 54.7)	0/14 (0%)

Table S2 Complete added value scoring per body part-specific PMMRI and indication

PMMRI per body part n=163	Indication	Additional finding (n, %, CI 95%)	Exclusion of a finding (n, %, CI 95%)	Confirmation of PMCT (n, %, CI 95%)	No added value (n, %, CI 95%)
**PMMRI brain**	*Total (n=52)*	19/52 (36.5%, 24.8 – 50.1)	6/52 (11.5%, 5.4 – 22.9)	18/52 (34.6%, 23.2 – 48.2)	16/52 (30.8%, 19.9 – 44.3)
	Suspected strangulation (n=25)	4/25 (16.0%, 6.4 - 34.7)	2/25 (8.0%, 2.2 – 24.9)	6/25 (24.0%, 11.5 – 43.4)	13/25 (52.0%, 33.5 – 69.9)
	PMMRI based on PMCT (n=15)	10/15 (66.7%, 41.7 – 84.8)	3/15 (20.0%, 7.1 – 45.2)	7/15 (46.7%, 24.8 – 69.9)	-
	Blunt force trauma (n=8)	4/8 (50.0%, 21.5 – 78.5)	1/8 (12.5%, 2.2 – 47.1)	4/8 (50.0%, 21.5 – 78.5)	1/8 (12.5%, 2.2 – 47.1)
	Penetrating injury (n=4)	1/4 (25.0%, 4.6 – 69.9)	-	1/4 (25.0%, 4.6 – 69.9)	2/4 (50.0%, 15.0 – 85.0)
**PMMRI neck**	*Total (n=63)*	22/63 (34.9%, 24.3 – 47.3)	19/63 (30.2%, 20.2 – 42.4)	25/63 (39.7%, 28.5, 52.0)	8/63 (12.7%, 6.6 – 23.1)
	Suspected strangulation (n=46)	15/46 (32.6%, 20.9 – 47.0)	16/46 (34.8%, 22.7 – 49.2)	17/46 (37.0%, 24.5 – 51.4)	5/46 (10.9%, 4.7 – 23.0)

	Penetrating injury (n=9)	4/9 (44.4%, 18.9 – 73.3)	2/9 (22.2%, 6.3 – 54.7)	3/9 (33.3%, 12.0 – 64.6)	3/9 (33.3%, 12.0 – 64.6)
	PMMRI based on PCMT (n=4)	2/4 (50.0%, 15.0 – 85.0)	1/4 (25.0%, 4.6 – 69.9)	2/4 (50.0%, 15.0 – 85.0)	-
	Blunt force trauma (n-3)	-	-	3/3 (100%, 43.9 - 100)	-
	Suspected oesophageal injury (n=1)	1/1 (100%, 20.7 - 100)	-	-	-
**PMMRI thorax**	*Total (n=31)*	22/31 (71.0%, 53.4 – 83.9)	6/31 (19.4%, 9.2 – 36.3)	4/31 (12.9%, 5.1 – 28.9)	3/31 (9.7%, 3.4 – 24.9)
	Penetrating injury (n=20)	14/20 (70.0%, 48.1 – 85.4)	3/20 (15.0%, 5.2 – 36.0)	3/20 (15.0%, 5.2 – 36.0)	3/20 (15.0%, 5.2 – 36.0)
	PMMRI based on PMCT (n=8)	6/8 (75.0%, 40.9 – 92.9)	3/8 (37.5%, 13.7 – 69.4)	-	-
	Blunt force trauma (n=2)	1/2 (50.0%, 9.5 – 90.6)	-	1/2 (50.0%, 9.5 – 90.6)	-
	Suspected oesophageal injury (n=1)	1/1 (100%, 20.7 - 100)	-	-	-
**PMMRI abdomen**	*Total (n=16)*	6/16 (37.5%, 18.5 – 61.4)	4/16 (25.0%, 10.2 – 49.5)	4/16 (25.0%, 10.2 – 49.5)	2/16 (12.5%, 3.5 – 36.0)

	Penetrating injury (n=9)	4/9 (44.4%, 18.9 – 73.3)	1/9 (11.1%, 1.9 – 43.5)	3/9 (33.3%, 12.1 – 64.6)	1/9 (11.1%, 1.9 – 43.5)
	PMMRI based on PMCT (n=5)	2/5 (40.0%, 11.8 – 76.9)	2/5 (40.0%, 11.8 – 76.9)	-	1/5 (20.0%, 3.6 – 62.5)
	Suspected early pregnancy (n=2)	-	1/2 (50.0%, 9.4 – 90.6)	1/2 (50.0%, 9.4 – 90.6)	-
**PMMRI extremities**	*Total (n=1)*				
	PMMRI based on PMCT: vascular injury	-	-	1/1 (100%, 20.7 - 100)	-
